# Bone Regeneration in Artificial Jaw Cleft by Use of Carbonated Hydroxyapatite Particles and Mesenchymal Stem Cells Derived from Iliac Bone

**DOI:** 10.1155/2012/352510

**Published:** 2012-03-26

**Authors:** Motoko Yoshioka, Kotaro Tanimoto, Yuki Tanne, Keisuke Sumi, Tetsuya Awada, Nanae Oki, Masaru Sugiyama, Yukio Kato, Kazuo Tanne

**Affiliations:** ^1^Department of Orthodontics and Craniofacial Developmental Biology, Hiroshima University Graduate School of Biomedical Sciences, 1-2-3 Kasumi, Minami-ku, Hiroshima 734-8553, Japan; ^2^Department of Oral Health Sciences, Hiroshima University Graduate School of Biomedical Sciences, 1-2-3 Kasumi, Minami-ku, Hiroshima 734-8553, Japan; ^3^Department of Dental and Medical Biochemistry, Hiroshima University Graduate School of Biomedical Sciences, 1-2-3 Kasumi, Minami-ku, Hiroshima 734-8553, Japan

## Abstract

*Objectives of the Study*. Cleft lip and palate (CLP) is a prevalent congenital anomaly in the orofacial region. Autogenous iliac bone grafting has been frequently employed for the closure of bone defects at the jaw cleft site. Since the related surgical procedures are quite invasive for patients, it is of great importance to develop a new less invasive technique. The aim of this study was to examine bone regeneration with mesenchyme stem cells (MSCs) for the treatment of bone defect in artificially created jaw cleft in dogs. *Materials and Methods*. A bone defect was prepared bilaterally in the upper incisor regions of beagle dogs. MSCs derived from iliac bone marrow were cultured and transplanted with carbonated hydroxyapatite (CAP) particles into the bone defect area. The bone regeneration was evaluated by standardized occlusal X-ray examination and histological observation. *Results*. Six months after the transplantation, perfect closure of the jaw cleft was achieved on the experimental side. The X-ray and histological examination revealed that the regenerated bone on the experimental side was almost equivalent to the original bone adjoining the jaw cleft. *Conclusion*. It was suggested that the application of MSCs with CAP particles can become a new treatment modality for bone regeneration for CLP patients.

## 1. Introduction

Cleft lip and palate (CLP) is a prevalent congenital anomaly in the orofacial region characterized by a jaw cleft due to failure of the palatal shelves to fuse properly. CLP is caused by various genetic and environmental factors, and the incidence rate of CLP is 0.19% in Japan [1]. Generally, CLP patients receive labiaplasty and initial palatoplasty at the infant stage as the initial treatment, and speech therapy is also needed for the recovery of speech function. In addition, discontinuity of the upper dental arch due to the jaw cleft frequently causes malocclusion, and orthodontic treatment is conducted on most CLP patients for acquisition of stable occlusion.

 In 1972, Boyne and Sands [2] reported that smooth eruption of the canine to the bone transplant area was induced and excellent dental arch form was obtained by autogenous iliac bone grafting before canine eruption. Since then, autogenous iliac bone grafting has been frequently employed for the closure of bone defects at the jaw cleft site to establish well-balanced occlusion [3–5].

 However, the related surgical procedures to collect transplant material from iliac bone are quite invasive, causing large stress for the patients. Therefore, artificial transplant materials for bone regeneration instead of iliac bone, such as hydroxyapatite (HAP) and *β*-tricalcium phosphate (*β*-TCP), have been suggested [6, 7]. To date, general clinical application of these materials alone has been limited because of the difficulty in induction of canine eruption and tooth movement to the transplant area [6, 7]. Therefore, it is of great importance to develop a new less invasive technique.

 Tissue engineering with stem cell transplantation is an expected candidate to achieve osteogenesis in bone defects with less surgical invasion for ilium extraction. Recently, stem cells have been applied to regenerative medicine, even for internal organs such as blood vessels, nerves, and heart [8–10]. For bone regeneration, mesenchyme stem cells (MSCs) have been expected to become useful transplant material. MSCs account for 0.001~0.01% of the subcellular component in bone marrow, having the potential to differentiate into multiple mesenchyme lineages such as chondrocytes, adipocytes, and osteoblasts by appropriate biological stimuli [11]. Sufficient volume of bone marrow for separation of MSCs can be aspirated using a bone marrow puncture needle. The pain score was significantly lower in CLP patients who underwent bone marrow puncture from iliac bone than in those who underwent conventional surgical separation of iliac bone marrow, suggesting that the bone regeneration using MSCs can relieve stress of patients [12]. However, there have been few studies on osteogenesis with MSCs in CLP patients [13, 14].

 Three elements (cell, scaffold, and growth factor) are believed to be crucial for successful tissue regeneration. In the present study, we used carbonated hydroxyapatite (CAP) particles as a scaffold. CAP contains 3~5% carbonate ions by substitution in the HAP lattice structure and is the major mineral constituent of bone and teeth. Therefore, CAP scaffold has quite high biocompatibility with the body, suggesting a big advantage as a scaffold material.

 In this study, we examined bone regeneration using MSC transplantation with CAP scaffold for artificial bone defect.

## 2. Materials and Methods

### 2.1. Preparation of Artificial Jaw Cleft in Beagle Dogs

*Three-month-old female beagle dogs were used (N* = 3*). Permission for a series of experiments in this study was granted by the Animal Experiment Committee of Hiroshima University*. Briefly, the upper third incisors on both sides were extracted under general anesthesia (Somnopentyl, Kyoritsu, Tokyo, Japan). The alveolar and palatal bones were ground by about 5 mm in width and 10 mm in length to create bilateral bone defects. The osteoepiphyses were epibolied by suturing mucosa. Antibiotic (Baytril, Bayer HealthCare, Tokyo, Japan) was used to prevent infection before and after the surgery. After a 1-month healing period, the artificial jaw cleft was evaluated by X-ray imaging to check whether or not the bone defect was filled with bone by spontaneous recovery.

### 2.2. Isolation of MSCs and the Culture

During the healing period, bone marrow MSCs were isolated from the iliac bone of each dog and cultured. The bone marrow was aspirated with a bone marrow puncture needle (Taiyu Medical Co., Tokyo, Japan) from the iliac bone of beagle dogs under general anesthesia with pentobarbital (Somnopentyl, Kyoritsu). MSCs were seeded at a density of 5 × 10^5^ cells per 100 mm cell-culture dish (Corning, New York, NY, USA) in Dulbecco's modified Eagle's medium (DMEM, Sigma-Aldrich, St. Louis, MO, USA) supplemented with 10% fetal bovine serum (FBS; Biowest, Nuaillé, France), 10% NaHCO_3_, 0.7 *μ*g/mL L-glutamine, and antibiotics, under 5% CO_2_ atmosphere in a humidified incubator at 37°C. The medium was changed every other day, and the MSCs were subcultured until confluence. The second-passaged cells were used in all experiments.

### 2.3. Transplant Body

MSCs were cultured to 1 × 10^8^ cells/well and detached from the culture plates *just before transplantation. Unsintered CAP particles (600–800 *μ*m) containing about 10% carbonate ions were used as scaffold. One hundred and eighty mg CAP particles were mixed with MSCs to allow the cells to attach on the surface of CAP particles*. A transplant *consisting* of the same volume of CAP particles without MSCs was used as the control.

### 2.4. Transplantation of MSCs into Artificial Jaw Cleft

*One month after the preparation of artificial jaw cleft, the artificial jaw cleft on the both sides was opened under general anesthesia with pentobarbital (Somnopentyl, Kyoritsu)*. MSCs with CAP particles were transplanted into the bone defect on the left (experimental side), whereas transplant *consisting* of CAP particles alone was filled on the right side cleft (control side). The bone defect filled with a transplant was covered with poly-lactic-co-glycolic-acid (PLGA) barrier membrane (GC membrane, GC Co., Tokyo, Japan) to prevent leakage of the transplant. Afterward, mucous membrane was sutured to intimacy. Antibiotic (Baytril, Bayer HealthCare) was used to prevent infection before and after the surgery.

### 2.5. Evaluation of Bone Regeneration by X-Ray Imaging

**Figure 1:**
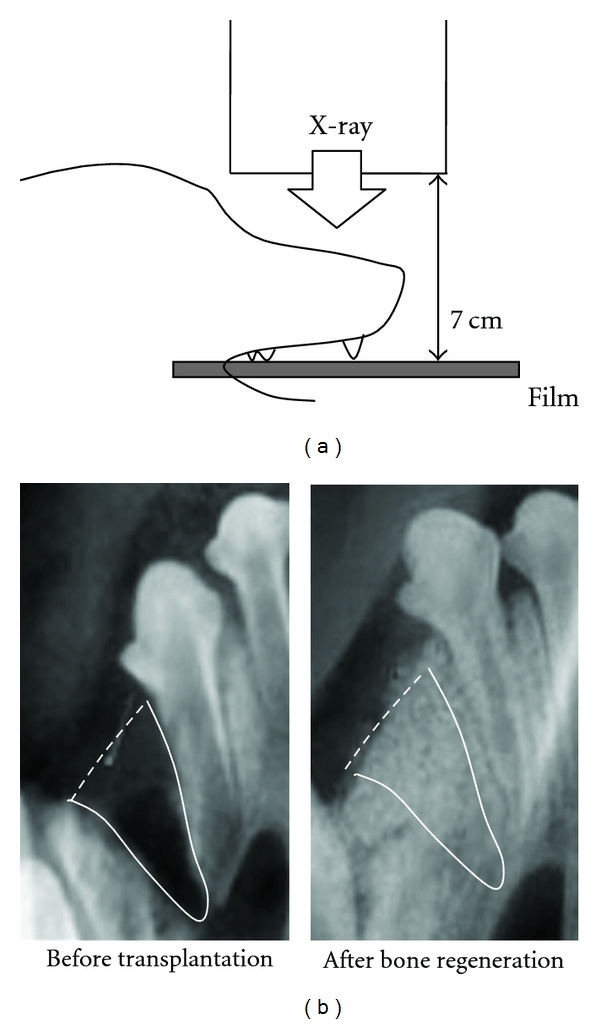
X-ray examination of bone regeneration (a) Schema of standardized occlusal X-ray examination. A film was held parallel to the upper dentition of a beagle dog, and X-rays were irradiated vertically to the film at 250 mV. The angle and distance between X-ray bulb and film were standardized using a locator. (b) The radio-opacity without signals of CAP particles in the artificial jaw cleft area was measured on standardized occlusal X-ray images.

Bone regeneration was evaluated using standardized occlusal X-ray images and histological examination. All occlusal X-ray examinations in this study were standardized using a film holder to maintain positions among X-ray irradiator, object, and film (Figure 1(a)).

The radio-opacity of the artificial jaw cleft area was measured using *NIH-image* 1.59 software (National Institutes of Health, Bethesda, Washington DC, USA) on the standardized occlusal X-ray images (Figure 1(b)). *The signals of regenerated bone and CAP particles were evaluated by radio-opacity of the jaw cleft area*.

### 2.6. Evaluation of Bone Regeneration by Histological Staining

*A small piece of the regenerated tissue was separated from the dogs under general anesthesia with pentobarbital (Somnopentyl, Kyoritsu) 3 and 6 months after the transplantation*. The tissue specimens were immediately fixed with 4% paraformaldehyde in phosphate-buffered saline (PBS) for 5 min, decalcified with EDTA for 1 month, and embedded in paraffin. Tissue sections of 7 *μ*m thickness were made and stained with hematoxylin and eosin (HE).

 The number of capillary vessels in the regenerated area was counted on the tissue sections using a phase contrast microscope (BZ8000, KEYENCE, Osaka, Japan).

### 2.7. Statistical Analysis

*The transplantation of MSCs was performed using 3 dogs*. Means and standard deviations (SD) were calculated from the data obtained and then subjected to Student's *t*-test using Graphpad Prism 4.0a software (Graphpad Software, Inc., San Diego, CA, USA) to examine significant differences in the means at the 1% and 5% levels of significance.

## 3. Results

### 3.1. Intraoral Findings before and after the Transplantation of MSCs to Artificial Jaw Cleft

**Figure 2:**
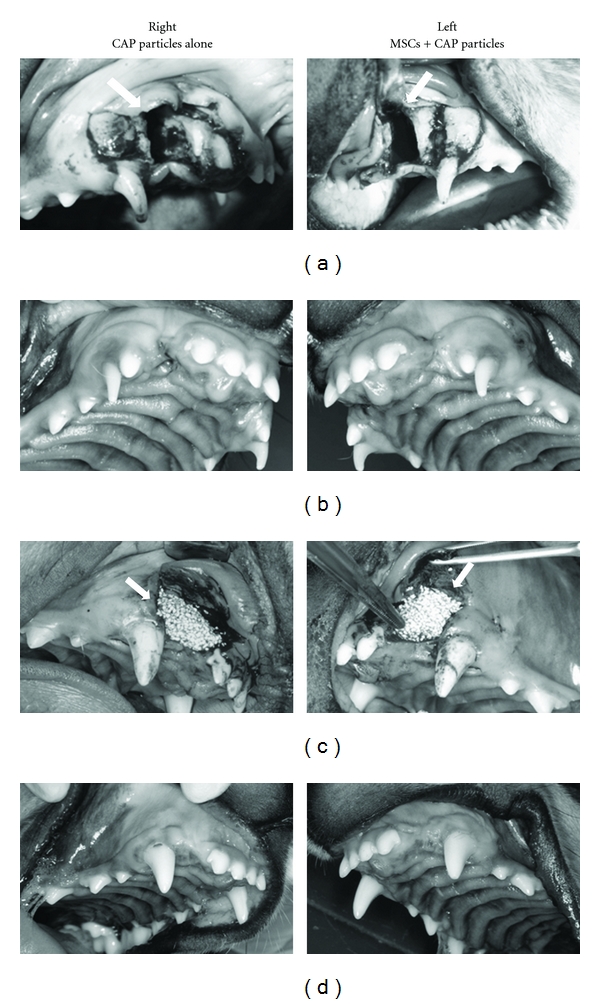
Intraoral findings before and after the transplantation of MSCs to artificial jaw cleft. Intraoral images (a) after preparation of bilateral artificial jaw cleft in maxilla (arrowhead, jaw cleft), (b) before and (c) after transplantation of MSCs with CAP particles to the jaw cleft (3 months after the preparation of artificial jaw cleft). The cleft on the right side (control side) was filled with CAP particles alone, whereas the cleft on the left side (experimental side) was filled with MSCs and CAP particles (arrowhead). (d) 6 months after the transplantation.

Bilateral artificial jaw cleft was prepared on the dog maxilla (Figure 2(a), arrowhead). The artificial jaw cleft was opened after 3 months, and no spontaneous recovery of bone defect was shown. Thus, the CAP particles with or without MSCs were transplanted to the bone defects (Figure 2(c), arrowhead). After the transplantation, no inflammation was shown in the transplanted area. Six months after the transplantation, the shape of the alveolar ridge at the transplanted area was maintained on both experimental and control sides (Figure 2(d)).

### 3.2. Radiographic Findings before and after the Transplantation of MSCs to Artificial Jaw Cleft

**Figure 3:**
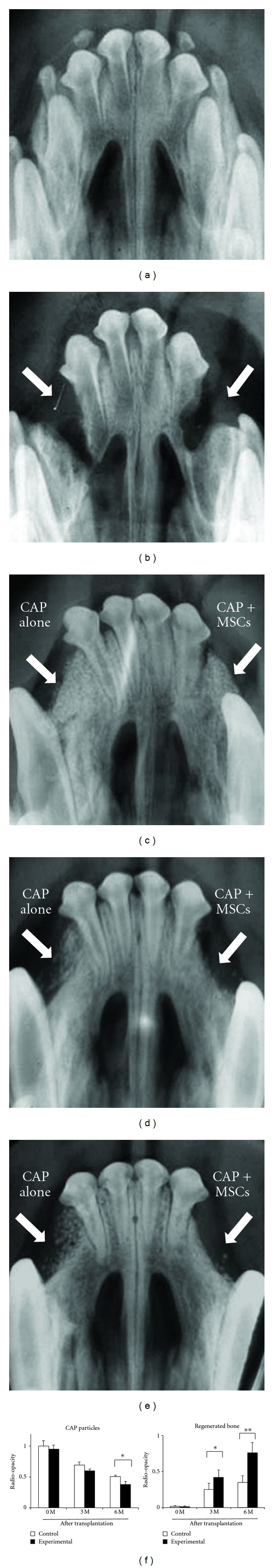
Radiographic findings before and after the transplantation of MSCs to artificial jaw cleft. Standardized occlusal X-ray images (a) after preparation of bilateral artificial jaw cleft in maxilla, (b) before and (c) after transplantation of MSCs with CAP particles to the jaw cleft (3 months after the preparation of artificial jaw cleft; arrowhead, jaw cleft). The cleft on the right side (control side) was filled with CAP particles alone, whereas the cleft on the left side (experimental side) was filled with MSCs and CAP particles, (d) 3 months and (e) 6 months after the transplantation (arrowhead, transplanted area). (f) The radio-opacity of the artificial jaw cleft area was measured on the standardized occlusal X-ray images after the transplantation to the artificial jaw cleft. *The signals of regenerated bone and CAP particles were evaluated by radio-opacity of the jaw cleft area*. *N* = 3, *P < 0.05, **P < 0.01.

No spontaneous recovery of bone defects was shown by X-ray examination 3 months after the preparation of bilateral artificial jaw cleft (Figures 3(a) and 3(b), arrowhead). Immediately after the transplantation, CAP particles were observed as opaque regions in images of the jaw cleft (Figure 3(c), arrowhead). Three months after the transplantation, radio-opacity of CAP particles was reduced on the control side compared with that on the experimental side (Figure 3(d), arrowhead), and decreased more substantially after 6 months (Figure 3(e), arrowhead). The radio-opacity on the experimental side after 6 months was increased whereas the number of CAP particles was decreased compared with those at 3 months.

*The radio-opacity of CAP particles on the experimental side was significantly ( P* < 0.05*) lower than that on the control side (Figure 3(f)). On the other hand, the radio-opacity of regenerated bone on the experimental side was significantly (3 months,* *P* < 0.05*; 6 months, P* < 0.01*) higher than that on the control side*. These results *indicate* the digestion of CAP particles and calcification in the jaw cleft on the experimental side.

### 3.3. Histological Observation before and after the Transplantation of MSCs to Artificial Jaw Cleft

Three and *Six* months after the transplantation, the tissues of the transplanted area were separated and evaluated by histological observation.

**Figure 4:**
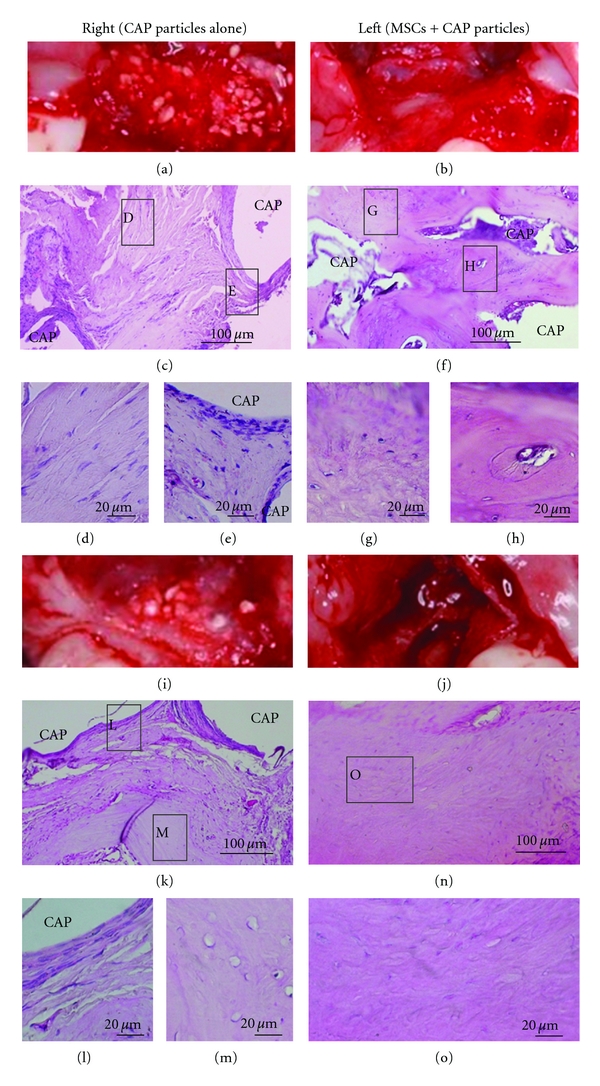
Histological observation before and after the transplantation of MSCs to artificial jaw cleft. The regenerated tissues were separated 3 and 6 months after the transplantation. Tissue sections were made and stained with hematoxylin and eosin (HE). Intraoral images 3 months after the transplantation on (a) the control side (CAP alone) and (b) the experimental side (MSCs and CAP particles). The tissue section showed fibroblastic cells and inflammatory cells around CAP particles on the control side (c, d, and e). New bone formation was shown widely on the experimental side (f, g, and h). Intraoral images 6 months after the transplantation on (i) the control side and (j) the experimental side. The tissue section showed that new bone formation had occurred locally on the control side (k, l, and m). On the other hand, new bone formation was observed in almost the whole area on the experimental side (n and o).

 Three months after the transplantation, a large *number* of CAP particles *remained* on the control side, whereas only a few CAP particles were found on the experimental side on the intraoral photographs (Figures 4(a) and 4(b)). In addition, the CAP particles had become smaller than the original particles (600–800 *μ*m) on the experimental side. Histological observation revealed the presence of fibroblastic cells around CAP particles on the control side (Figures 4(c), 4(d), and 4(e)). On the other hand, no fibroblastic cells were shown around the CAP particles, and new bone formation was observed on the *experimental* side (Figures 4(f), 4(g), and 4(h)).

 Six months after the transplantation, the number of CAP particles on the control side was decreased but many particles still remained, whereas almost no CAP particles were observed on the experimental side (Figures 4(i) and 4(j)). Histological examination revealed that new bone formation was present locally in the transplanted area on the control side, but fibroblastic cells were still located around CAP particles (Figures 4(k), 4(l), and 4(m)). On the other hand, new bone formation was observed in almost the whole area on the experimental side, and the CAP particles had almost disappeared (Figures 4(n) and 4(o)).

**Figure 5:**
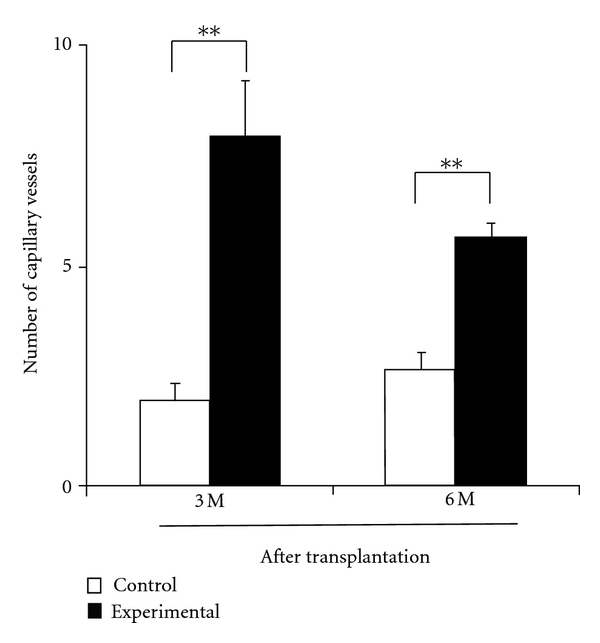
Number of capillary vessels in the regenerated area of artificial jaw cleft. Number of Capillary Vessels in Regenerated Area Was Counted on the Tissue Sections using a Phase Contrast Microscope. *N* = 3, **P < 0.01.

 The number of capillary *vessels* was significantly (*P* < 0.01) greater on the experimental side than on the control side after 3 and 6 months (Figure 5).

## 4. Discussion

In the present study, bone regeneration of artificial jaw cleft was demonstrated by the transplantation of MSCs with CAP particles. Radio-opacity of regenerated tissue on the experimental side was significantly higher than that on the control side, suggesting a contribution of MSCs to new bone formation. The CAP particles used in the present study *were* unsintered, and substituted for 3–5% carbonate ions in the HAP structure, leading to unstable crystal structure compared with that of HAP. Since the solubility of pure HAP is quite low, it would take a long time to be digested and replaced *by* new bone if used for transplantation into bone defect. In a previous study [15], CAP transplanted above a tooth bud did not disturb tooth eruption, suggesting its high biocompatibility and solubility in the body. However, there have been no reports of bone regeneration in jaw cleft by the use of unsintered CAP scaffold. In our previous study, alveolar bone regeneration was achieved by transplantation of *β*-TCP in mouse [6]. However, the scaffold for CLP alveolar bone regeneration requires some special characteristics. *The transplantation of *β*-TCP was suggested to cause disturbance of canine eruption and tooth movement after its transplantation [6]*. Generally, alignment of tooth on the jaw cleft area by tooth movement or transplantation is performed after bone regeneration in the treatment of CLP patients [3, 16]. Therefore, the jaw cleft should be filled with bone having normal metabolism to allow tooth eruption or movement. For this purpose, transplant material with high solubility in a living body and that can be digested quickly is considered to be suitable.

 In the present study, bone regeneration was accompanied by the absorption of CAP particles by the transplantation of CAP particles and MSCs on the experimental side. Meanwhile, absorption of CAP particles and new bone formation were much slower on the control side. In a living body, osteoclasts and osteoblasts at various differentiational phases act as one physiologic unit (basic multicellular unit; BMU), which controls bone remodeling repeatedly. The balance of bone absorption and formation is modulated by BMU activity, and new bone formation is activated after bone absorption. During the activation phase of bone remodeling, osteoclasts secrete acid and specific enzymes for continuous digestion of bone-salt and bone matrix proteins, respectively [17]. Therefore, it is speculated that digestion of transplant material plays a crucial role in new bone formation, and the difference in bone regeneration between the experimental and control sides may be due to the rate of CAP absorption in this study.

 In the present study, the number of capillary *vessels* in regenerated tissue after transplantation was significantly greater on the experimental side than on the control side. It was suggested that MSCs express vascular endothelial growth factor (VEGF), which is advantageous for bone regeneration by inducing vascularization [18]. Since the enhancement of vascularization is favorable to induce osteoclasts in the bone regeneration area, it is assumed that transplantation of MSCs may contribute to angiogenesis, leading to enhancement of CAP digestion by osteoclast induction. The transplantation of MSCs may have relevance for not only their differentiation ability into osteoblasts but also for induction of capillary vessels. If this is true, the transplantation of undifferentiated MSCs has an advantage compared with the application of differentiated MSCs to osteoblasts. However, it remains unclear whether transplanted MSCs differentiated into osteoblasts or just *expressed* cytokines or growth factors after transplantation. Tracing of transplanted MSCs would be beneficial to resolve the above issue in future studies.

 In addition, transplanted CAP particles on the control side were encapsulated with fibroblastic cells, and *this* may *be* another reason *for* the delay in digestion of CAP particles. However, it remains unclear why CAP particles on the experimental side were not encapsulated with fibroblastic cells. Therefore, the mechanism of CAP digestion should also be clarified in future studies.

 A specific requirement in bone regeneration of jaw cleft is *the* guidance or movement of tooth into newly formed bone. In this study, sufficient volume of alveolar bone for tooth movement was formed in the artificial jaw cleft by the transplantation of MSCs with CAP particles. Orthodontic tooth movement is a result of bone remodeling, which has high relevance to angiogenesis [19]. Since the regenerated tissue on the control side has a small number of blood vessels as well as undigested CAP particles, difficulty of orthodontic tooth movement was speculated to be due to low bone remodeling.

 In conclusion, it was shown that the transplantation of MSCs with CAP particles could regenerate bone in artificial jaw cleft. The transplantation of MSCs with CAP particles to jaw cleft may be a new treatment modality for CLP patients.
